# Benchmark Development for Fundamental Arthroscopic Skills Using a Simulation-Based Training Program: Observational Study

**DOI:** 10.2196/82723

**Published:** 2026-04-08

**Authors:** Eric Davis, Brianna Caraet, Robert Pedowitz, Gregg Nicandri

**Affiliations:** 1 Department of Orthopaedic Surgery, University of Rochester, 601 Elmwood Avenue, Rochester, NY, 14642, United States, 1 585-276-4874; 2 Kaiser Permanente Orthopedic Sports Medicine, San Diego, CA, United States; 3 Department of Orthopaedic Surgery, University of California, Los Angeles, CA, United States

**Keywords:** surgical education, orthopedic surgery, arthroscopy, simulation training, skills assessment, resident education

## Abstract

**Background:**

Surgical education has shifted from the traditional Halstedian apprenticeship model toward incorporating simulation due to work-hour restrictions, increasing case complexity, and economic and liability pressures. Building on the success of the Fundamentals of Laparoscopic Surgery program for general surgery, the Fundamentals of Arthroscopic Surgery Training (FAST) program was developed to establish proficiency benchmarks for orthopedic trainees in basic arthroscopic skills.

**Objective:**

We aimed to establish benchmarks for 5 FAST workstation modules.

**Methods:**

Sports medicine fellowship–trained faculty members were given instructions on the modules and 2 minutes of practice time, and they then performed each task 3 times with both their dominant and nondominant hand. For each module, mean faculty performance was used to establish an efficiency benchmark (time) and precision benchmark (errors).

**Results:**

The Probing module should be completed in less than 95 seconds with no errors. The Ring Transfer module should be completed in less than 134 seconds with no more than 1 error. The Maze module should be completed in less than 99 seconds with no errors. The Meniscectomy module should be completed in less than 68 seconds with no more than 1 error. Lastly, the Suture Passing module should be completed in less than 195 seconds with no more than 1 error.

**Conclusions:**

The FAST workstation can be used as a proficiency-based learning tool for residents to safely and effectively develop arthroscopic skills outside of the operating room. These benchmarks were established via a method previously validated in surgical simulation and balance precision and efficiency for skills that are considered generalizable and transferable to arthroscopic surgeries.

## Introduction

Historically, surgical education has been based upon the Halstedian apprenticeship model, in which surgical trainees primarily acquire technical skills in the operating room while assisting and later performing procedures on live patients. In recent years, there has been a paradigm shift away from this approach in orthopedic surgery [1]. This can be attributed to multiple factors, including but not limited to resident work-hour restrictions, increased case complexity and variety, the risk of liability, and social and economic pressure. Simulation in surgical education has been suggested as a potential avenue to fill the gap in training, allowing trainees opportunities to obtain valuable experience in a low-risk, more cost-effective environment. In concordance with this, the Accreditation Council for Graduate Medical Education now requires that residency programs include surgical skills training in their curricula; this mandate has also been echoed by the American Board of Orthopaedic Surgery and the Residency Review Committee.

Simulation as an educational tool has been validated extensively in the field of general surgery. The Fundamentals of Laparoscopic Surgery program, developed in 1997 by the Society of American Gastrointestinal and Endoscopic Surgeons, combines cognitive and physical skills components shown to improve a given student’s laparoscopic abilities over the course of the curriculum [2]. The program has been studied rigorously and validated since its release [3-9]. Currently, a passing score is required by the American Board of Surgery as prerequisite to sit for the certification exam. This has been well received by trainees, surgeons, and the public. As a result of this successful program in general surgery, the field of orthopedics has explored the development of a similar program.

In 2011, the Arthroscopy Association of North America (AANA), the American Board of Orthopaedic Surgery, and the American Academy of Orthopaedic Surgeons came together in a joint effort to establish the Fundamentals of Arthroscopic Surgery Training (FAST) program. The program is based on the tenet that fundamental surgical skills are best obtained sequentially, and that proficiency can be established through the successful completion of a basic skills curriculum. A curriculum and simulator have been developed for the FAST program and proficiency benchmarks for knot tying have been established.

The aim of this study was to develop time- and error-based proficiency benchmarks for 5 fundamental arthroscopic skills modules within a simulation-based training program.

## Methods

### Overview

The AANA offered a voluntary skills training course for residents and faculty at the Orthopaedic Learning Center in Chicago, Illinois, on December 7 to 9, 2017, and January 18 to 20, 2018. During downtime in the course, participants were recruited and provided with an information sheet outlining participation in this study. Two separate cohorts of 20 faculty each completed orthopedic simulations to improve their skill. For each module, the participant was given a list of written instructions and allowed to practice for a period of 2 minutes. Then, each participant performed the task 3 times with their dominant hand and 3 times with their nondominant hand. The order of dominant/nondominant completion was randomized so that exactly half of the faculty started with their nondominant hand. The time it took each participant to complete the module as well as the number of errors committed was recorded. Cohort 1 completed the Probing, Ring Transfer, and Maze Navigation modules. Cohort 2 completed the Meniscectomy and Suture Passing modules.

All modules were completed using camera-based visualization rather than live arthroscopes. Depending on the module, visualization was provided using either a USB camera system or a stationary camera, consistent with the simulation equipment available in the skills laboratory. This approach was used to standardize task execution across participants and to support reproducibility in a controlled training environment.

Five simulation-based arthroscopic skills modules were evaluated, including Probing, Ring Transfer, Maze Navigation, Partial Meniscectomy, and Suture Passage. Each module assessed fundamental psychomotor skills relevant to arthroscopy, with efficiency measured by task completion time and precision measured by predefined error criteria. Detailed descriptions of each module are provided in Multimedia Appendix 1.

Descriptive statistics were used for demographic variables, experience, handedness, efficiency (time) score, and precision (error) score. Mean performance time for all participants was determined. Any value lying more than 2 SDs from the mean was excluded. Exclusion of values greater than 2 SDs from the mean was performed to minimize the influence of extreme outliers that may reflect momentary lapses, equipment issues, or deviations from standardized task execution rather than true expert performance. This approach has been used in prior simulation-based benchmarking studies to derive stable proficiency thresholds that reflect typical expert performance rather than maximal or anomalous values. Outlier exclusion affected a small proportion of observations and did not meaningfully alter the relative ordering of module performance or the final benchmark thresholds. The trimmed mean was used as the basis for determining the proficiency level benchmark for each task. Efficiency benchmarks were set at the minimum threshold of the mean time of the expert cohort. Precision benchmarks were set at the mean number of errors committed by participants rounded to the nearest whole number. The benchmark was established as the mean performance when data from both hands were aggregated. Aggregation of dominant and nondominant hand performance was chosen a priori to reflect the ambidextrous demands of arthroscopic surgery, in which instrument laterality varies by procedure and portal placement. Use of the mean performance across both hands was intended to establish benchmarks that promote bilateral skill development while remaining feasible and generalizable for trainee progression.

### Ethical Considerations

This study was reviewed by the University of Rochester Research Subjects Review Board and was granted exempt status with a waiver of documentation of informed consent. All participants were provided with an information sheet outlining the purpose, procedures, and voluntary nature of the study prior to participation. All data collected were deidentified. Each participant was assigned a unique study identification number that contained no personally identifiable information. No names or direct identifiers were recorded at any point in data collection or analysis. To protect participant privacy and confidentiality, data were recorded using study IDs only and stored securely in accordance with institutional research guidelines. Only members of the research team had access to the data. Results are presented in aggregate form to prevent identification of individual participants. Participation in this study was voluntary. Participants were not provided with any financial compensation or incentives for participation, and there were no costs associated with participation.

## Results

### Demographics

A total of 40 sports medicine fellowship–trained faculty surgeons were included in the analysis. The mean age of the participants was 43.9 (SD 9.0) years. They had an average of 10.5 (SD 8.3) years in practice and performed approximately 277 (SD 125) arthroscopic cases per year. All were male and 3 (8%) were left-handed. There were no significant differences in demographic data between cohorts from the 2 courses (Table 1).

**Table 1.** Demographics.

	Cohort 1 (n=20)	Cohort 2 (n=20)	Overall (n=40)	*P* value^b^
Age, mean (SD)	43.1 (8.38)	44.7 (9.22)	43.9 (9.0)	.59
Male gender, n (%)	20 (100)	20 (100)	40 (100)	—^a^
Percent fellowship trained, n (%)	20 (100)	20 (100)	40 (100)	—
Years in practice, mean (SD)	9.5 (7.7)	11.4 (8.5)	10.5 (8.3)	.48
Cases per year (n), mean (SD)	275 (130.1)	279 (117.3)	277 (125)	.94
Right-handed, n (%)	18 (90)	19 (95)	37 (93)	.55

a Not applicable.

b For age, number of years in practice, and number of cases per year, 2-sided *t* tests were used to test for statistical significance. For handedness, the *χ*2 test was used. No differences were statistically significant.

### Probing

For the Probing module, mean completion times were 92.5 (SD 17.6) seconds for the dominant hand and 97.3 (SD 13.6) seconds for the nondominant hand, with no errors recorded and no significant difference between hands (*P*=.35); the benchmark was <95 seconds with no errors.

### Ring Transfer

For the Ring Transfer module, mean completion times were 117.5 (SD 26.7) seconds for the dominant hand and 151.3 (SD 38.1) seconds for the nondominant hand, with mean error rates of 0.6 (SD 0.8) and 1.1 (SD 0.9) dropped rings, respectively; dominant and nondominant hand performance differed significantly (*P*<.001), and the benchmark was <134 seconds with no more than 1 dropped ring.

### Maze

For the Maze module, mean completion times were 89.1 (SD 24.3) seconds for the dominant hand and 108.6 (SD 25.0) seconds for the nondominant hand, with mean error rates of 0.2 (SD 0.4) and 0.1 (SD 0.3) lost balls, respectively; dominant and nondominant hand performance differed significantly (*P*=.02), and the benchmark was <99 seconds with no errors.

### Meniscectomy

For the Meniscectomy module, mean completion times were 73.2 (SD 20.7) seconds for the dominant hand and 63.7 (SD 23.6) seconds for the nondominant hand, with mean error rates of 0.1 (SD 0.3) over- or underresection events for both hands; no significant difference was observed between dominant and nondominant hand performance (*P*=.14), and the benchmark was <68 seconds with no more than 1 area of over- or underresection.

### Suture Passing

For the Suture Passing module, mean completion times were 177.5 (SD 41.2) seconds for the dominant hand and 211.9 (SD 40.9) seconds for the nondominant hand, with mean error distances of 0.5 (SD 0.6) mm and 0.6 (SD 0.8) mm from the target, respectively; dominant and nondominant hand performance differed significantly (*P*<.001), and the benchmark was <195 seconds with no more than 1 mm from the target and no suture anchor unload.

For all modules, benchmark time was defined as the mean performance across dominant and nondominant hands to reflect bilateral task demands. For example, if the right hand took 60 seconds and the left hand took 30 seconds, the benchmark time was 45 seconds. Benchmarks for all modules are summarized in Table 2.

**Table 2.** Benchmarks.

Module	Benchmark time (seconds)^a^	Benchmark errors
Probing module	<95	No errors
Ring Transfer module	<134	No more than 1 dropped ring
Maze module	<99	No balls off platform
Meniscectomy module	<68	Only 1 area of over- or underresection
Suture Passing module	<195	<1 mm from the target area and no suture anchor unloads

a Benchmark time is the mean performance for both hands. For example, if the right hand takes 60 seconds, and the left hand takes 30 seconds, the time to compare against the benchmark is 45 (90/2) seconds.

## Discussion

### Principal Findings

In this study, we established objective time- and error-based proficiency benchmarks for 5 fundamental arthroscopic skills modules using performance data from fellowship-trained sports medicine faculty members. These benchmarks were derived using a standardized, previously validated simulation-based methodology and were designed to balance efficiency and precision across tasks of increasing technical complexity. Defining proficiency thresholds based on expert performance involves an inherent trade-off. Benchmarks that are too lenient may allow progression without adequate skill consolidation, whereas overly stringent thresholds may discourage practice or be impractical within residency training constraints. By deriving benchmarks from aggregated expert performance and excluding extreme outliers, the present thresholds were designed to balance feasibility with meaningful skill assessment. Continued evaluation of trainee outcomes and future transfer-validity studies will be important to further calibrate these benchmarks.

The FAST program consists of a curriculum, a simulator, and a series of tests, which enable trainees to iteratively practice fundamental skills outside of the operating room and ideally before performing surgery on patients. Training such motor skills outside of the OR is crucial in arthroscopy, which has a steep learning curve [10]. The FAST program’s hands-on design is in alignment with the Institute of Medicine’s recommendations for graduate medical education, which aim to transition from process-driven training to a more outcome-driven approach. Instead of the assumption that residents acquire their needed skills after completing a certain number of procedures or after a certain amount of time in training, the institute recommends evaluating proficiency in such skills before advancement [11]. The FAST program uses this proficiency-based progression approach to training to encourage “deliberate practice” to improve motor skills [12]. With the establishment of benchmarks for each FAST module, a resident is able to receive real-time objective feedback on their performance and is encouraged to strive to be comparable to experienced arthroscopy surgeons. With the accessibility of the workstations, residents are able to obtain additional practice repetitions and adjust their technique as needed to meet the established standard. Recent evaluations of the FAST workstation have demonstrated measurable differences in performance across postgraduate training levels and institutions, further supporting the need for standardized, objective benchmarks [13].

The FAST program can be integrated as early as the intern year in orthopedic training so that young surgeons can develop foundational psychomotor skills before even touching an arthroscope in the operating room. This early exposure to arthroscopic instruments and techniques can instill confidence in a new resident and their abilities, help them develop good habits early, and potentially decrease patient morbidity. Studies have demonstrated that medical students and residents who train with simulators perform better in the operating room than their counterparts without simulator training, with recent arthroscopy-specific work demonstrating transfer validity to diagnostic knee arthroscopy and meniscectomy performance [14,15]. With improved resident performance in the operating room, a faculty member walking a resident through the case would likely feel more comfortable increasing resident autonomy, and valuable operating room time can be spent teaching and instructing on the unique nuances of a particular case instead of on basic skills.

**Figure 1.**
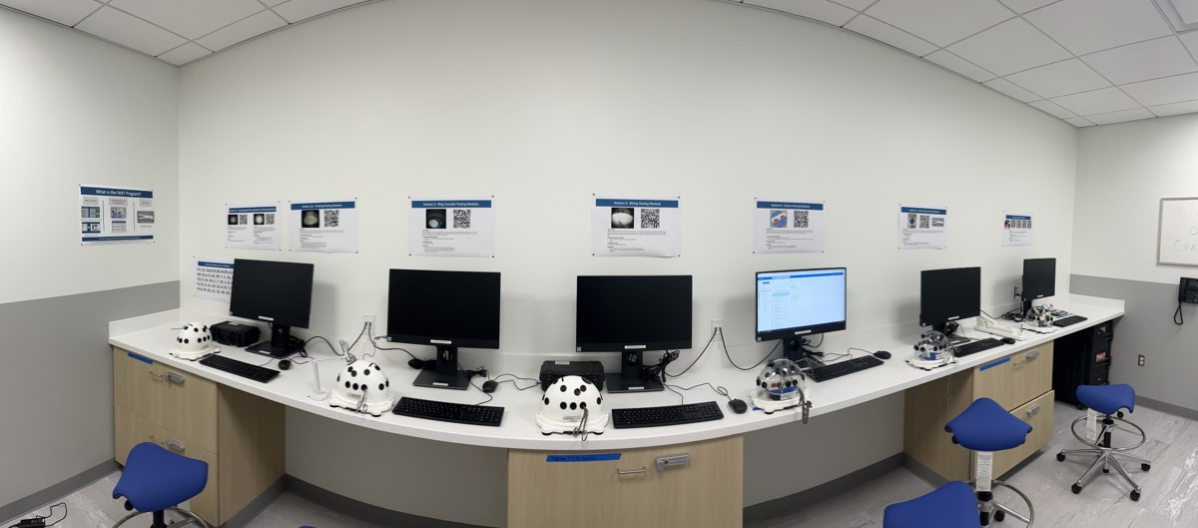
Simulation laboratory set-up.

The FAST workstations are easily accessible, cost-effective, and efficient. They require a simple set-up, and at our institution, they are available in the skills laboratory for all residents to use at any time. Residents can easily independently set up the workstation in the laboratory as long as they have an arthroscopy tower or USB camera with which to practice. Currently, we have each of the testing modules set up in front of a computer monitor with all the instruments available that are necessary to complete the task. We have a QR code that links to a video of the task and instructions mounted on the wall above each of the modules (Figure 1). We use iPevo cameras when a stationary light source is sufficient (Knot Tying) and Sawbones USB cameras when a replica of an arthroscope is required (Ring Transfer). We have found that having easy-to-access instructions and all modules already set up increases use of the simulator and maximizes resident time. We typically introduce FAST during a 2-to-3-hour session with all interns that is proctored by an instructor during their skills month. This familiarizes them with the simulator and modules. They are expected to practice to proficiency and then use the simulators to refine their skills prior to their arthroscopic rotations. The modular design of the FAST program also supports scalability across institutions with varying resource availability and learner profiles, as the workstations can be implemented using differing levels of visualization technology and integrated at multiple stages of training. This flexibility allows programs to adapt FAST implementation to local constraints while maintaining standardized proficiency benchmarks.

Prior papers have aimed to establish clear quantitative benchmarks for simulation training based on the mean performance of an experienced surgeon cohort [16]. One such paper, by Pedowitz et al [17], established the benchmark for the Knot Tying module in the FAST program, which is the last module to be completed in the series. It evaluated the performance of 50 faculty members in attendance at AANA resident arthroscopy courses. The 2 faculty cohorts had an average of 19.3 and 19.9 years of experience in practice. A benchmark was established using data from the more successful of the 2 faculty cohorts and established proficiency as equal to 2 knot failures or less out of 5 knot attempts when using the knot tester workstation. This randomized, prospective study then evaluated a training group of 44 postgraduate year 4 or 5 orthopedic residents. They were divided into 3 subgroups: group A received standard didactic training, group B was allowed additional knot-tying workstation practice, and group C received proficiency-based progression training with the knot-tying workstation. While the aggregate resident knot failure rate of 26% was higher than the 22% knot failure rate of the faculty, resident group C had only an 11% knot failure rate with 94% of residents in this group passing the threshold. This paper both demonstrated a system to establish a benchmark standard and proved that proficiency-based progression training improves the likelihood of meeting said benchmarks. Recent FAST-based educational interventions have demonstrated improvements in objective performance metrics, including arthroscopic knot integrity, when guided, proficiency-based training is used [18]. The arthroscopic knot-tying station is the last in the sequence of the FAST workstation modules as it tests one of the more complex skills in arthroscopy. The purpose of the current investigation was to establish objective benchmarks for the remaining 5 FAST workstation modules: Probing, Ring Transfer, Maze Navigation, Meniscectomy, and Suture Passing.

These benchmarks derived from the performance of fellowship-trained sports medicine faculty members were designed to be both realistic and achievable for residents. The 6 modules progress from basic skills to more complex tasks. The first module, Probing, allows residents to establish basic arthroscopic skills of horizontal control, telescoping, periscoping, and triangulation in order to feel comfortable performing a diagnostic arthroscopy. Gaining familiarity with the arthroscope in this module lays the foundation for establishing visualization in the remainder of the tasks and in the operating room. The second module, Ring Transfer, reinforces these skills and allows the resident to develop proficiency with the arthroscopic grasper, which is used when they are tasked with performing an arthroscopic loose-body removal. The dexterity with the instruments developed in this module is applicable to using arthroscopic instruments to manipulate tissues or implants in all arthroscopic procedures. The next module, Maze Navigation, builds on prior probing skills from the first module and develops tracking skills with nonstationary objects. This skill is necessary to develop before interventional arthroscopy can be performed. While the first 3 modules focus more on skills generalization, the final 3 modules correlate even more closely with specific surgical interventions, thereby focusing more on skills transfer. The fourth module directly simulates completing a partial meniscectomy with a benchmark designed to encourage an adequate amount of resection. During this practice, residents develop more familiarity with a biter and the ability to maneuver more deliberately and precisely. Resection of a paper “meniscus” is a safer alternative to a new resident damaging cartilage with the biter or over-resecting and destabilizing a meniscus tear because they are using a biter for the first time in an actual patient’s knee. The second to last task, Suture Passing, integrates the use of other new instruments, including an antegrade suture passer, a piercing suture passer/retriever, and a suture lasso or suture shuttle. This task’s surgical correlate is an arthroscopic rotator cuff repair or Bankart repair, which are among the more complex arthroscopic procedures. Completion of this task relies on the mastery and coordination of skills obtained in the previous modules. The last module, Knot Tying, allows residents to evaluate the biomechanical integrity of their arthroscopic knots in a setting safer than discovering loose knots in a failed arthroscopic suture repair. In conclusion, a resident’s ability to meet the time constraints and limit their errors for each task translates into more efficient and accurate performance in the operating room, which promotes patient safety.

In establishing the benchmarks, there was a statistically significant difference in performance between the faculty surgeons’ dominant and nondominant hands with the Ring Transfer, Maze, and Suture Passing modules. However, surgeons have to be ambidextrous. Which hand holds the arthroscope and which holds the remaining instruments is often based on the laterality of the procedure. Previous studies have shown that experienced arthroscopic surgeons are more ambidextrous than novices [19]. Specifically, experts demonstrate significantly smaller dominant-nondominant differences in task completion time and error rates on simulated arthroscopic tasks compared with novices, supporting bilateral performance assessment as a marker of skill acquisition. Therefore, the FAST program requires all tasks to be completed with the right hand and the left hand in separate attempts in order to develop skills in both hands regardless of hand dominance. Establishing separate benchmarks for the dominant and the nondominant hand based on the differences seen in the expert cohorts was considered. Aggregated performance metrics are commonly used in established simulation benchmarking frameworks, such as the Objective Structured Assessment of Technical Skill (OSATS) and the Fundamentals of Laparoscopic Surgery program, which define proficiency using composite measures rather than limb-specific performance. This supports the use of bilateral performance means when establishing reproducible and generalizable training benchmarks. That said, the scientific ideal needs to be weighed equally with practicality in this case. The FAST Program puts multiple new demands on trainees and faculty in the programs that adopt it. Ultimately, in order to be successfully adopted, it needs to be easy to implement. Based upon our experience facilitating many FAST courses at the Orthopaedic Learning Center in Chicago and at multiple residency programs, we determined that a single benchmark for each task was necessary.

We also discussed whether to take the mean performance of both hands or to set the benchmark at the level of the dominant hand. We chose the mean performance. This was the best compromise between ensuring adequate ambidexterity while also recognizing that differences exist, even in expert faculty members. The FAST program is designed to enhance surgical skills and promote deliberate, repetitive practice for benchmarks. We were concerned that setting the benchmark as the performance of the dominant hand of the faculty members would have made it significantly harder to achieve for the trainees. The goal was for residents to be able to pass the FAST program by the end of their third year in training, and we decided that the likely significant increase in repetitions required to meet the more stringent benchmark would be frustrating and time consuming for residents who already have many other competing demands. Passing the FAST program at the end of the third year does not mean that residents are proficient at arthroscopy. It means that they have sufficiently practiced and likely have improved their fundamental arthroscopic skills by meeting a minimum threshold of performance. They still have additional years in residency to build upon the skills acquired in the FAST program. Future validation of FAST benchmarks could be pursued through prospective randomized or longitudinal studies comparing proficiency-based FAST training with standard curricula. Studies should be powered to detect differences in operative performance and stratified by postgraduate year to assess differential benefit by training stage. Translation to operating room performance may be measured using objective metrics such as task-specific error rates, time to task completion, and validated global rating scales (eg, OSATS) during comparable arthroscopic procedures. This benchmarking is foundational work that is required to enable such an investigation.

The development of the FAST program benchmarks is one of the factors that allow this to be a self-sufficient progression learning tool, setting it apart from other skills programs. In 2007, the Carolinas Medical Center integrated a laparoscopic skills curriculum into their general surgery residency program [20]. A skills laboratory coordinator needed to be present to record the duration of participation in each task and keep track of errors. In contrast, the FAST program is designed as a web-based program residents can access independently at a time convenient for them. A stopwatch is built into the program to record time to complete a task, and each task has a section to input the number of errors. These factors determine progression to the next module, all of which can be done at the resident’s own pace. The paper about the Carolinas Medical Center program by Stefanidis et al [20] describes feedback occurring after each training session or when a resident is called in by the coordinator. With the FAST program, instant feedback is available with the clear benchmarks established for each task.

### Limitations and Future Studies

A limitation of this study was that the included participants were exclusively faculty members teaching at the AANA Fundamentals of Arthroscopic Surgery Residents Course. All participants were male, sports medicine fellowship–trained faculty members, which may limit generalizability across genders, subspecialties, and training environments. Ultimately, this was a convenience sample due to the significant logistical hurdles to obtaining data with another method from a large number of expert orthopedic surgeons. This may have introduced a selection bias for surgeons, and they may not be fully representative of a wider population of sports fellowship–trained surgeons. The included cohort were deemed experts by virtue of their completed training, but there were outliers in performance among the participants. There were 21 (of 320) outlier data points, and these were subsequently excluded from the dataset before formulating the benchmarks.

Another limitation of this study is that it did not demonstrate the feasibility of the FAST program or its transfer validity, necessitating further studies. Further investigation is also needed to determine the average number of repetitions and practice time required to meet the proficiency benchmarks. These results could then be subanalyzed based on training level. This would provide a clearer estimated duration of FAST programs for other orthopedic residency programs interested in integrating the FAST program into their curricula. Further application of the FAST program could also be used to assess whether those who reach proficiency benchmarks perform better than those with no simulation training on other simulated surgical procedures and in the operating room for comparable surgical procedures. Lastly, further studies are needed to investigate which subset of residents achieve the most benefit from the FAST program as determined by the greatest improvement in arthroscopic skills. This could be used to determine in which year of residency training the FAST program should be integrated.

Benchmarks in this study were derived on a module-specific basis using different expert cohorts and should be interpreted as reference values for individual skills rather than curriculum-level proficiency standards. This design limits direct cross-module comparison but reflects practical constraints of expert data collection and supports initial benchmark development for discrete arthroscopic tasks. Evaluation of learners across the full FAST curriculum will be necessary to assess proficiency progress and program-level validity.

### Conclusions

FAST workstations can be used as proficiency-based learning tools for residents to safely and effectively develop arthroscopic skills outside of the operating room. These benchmarks were established via a method previously validated in surgical simulation and balance precision and efficiency for skills that are considered generalizable and transferable to arthroscopic surgeries.

## Supplementary material

10.2196/82723 Multimedia Appendix 1 Detailed procedural descriptions.

## Abbreviations

AANA: Arthroscopy Association of North America
FAST: Fundamentals of Arthroscopic Surgery Training
OSATS: Objective Structured Assessment of Technical Skill

## References

[1] Pedowitz RA, Marsh JL (2012). Motor skills training in orthopaedic surgery: a paradigm shift toward a simulation-based educational curriculum. J Am Acad Orthop Surg.

[2] Derossis AM, Fried GM, Abrahamowicz M, Sigman HH, Barkun JS, Meakins JL (1998). Development of a model for training and evaluation of laparoscopic skills. Am J Surg.

[3] McCluney AL, Vassiliou MC, Kaneva PA (2007). FLS simulator performance predicts intraoperative laparoscopic skill. Surg Endosc.

[4] Soper NJ, Fried GM (2008). The fundamentals of laparoscopic surgery: its time has come. Bull Am Coll Surg.

[5] Scott DJ, Bergen PC, Rege RV (2000). Laparoscopic training on bench models: better and more cost effective than operating room experience?. J Am Coll Surg.

[6] Seymour NE, Gallagher AG, Roman SA (2002). Virtual reality training improves operating room performance: results of a randomized, double-blinded study. Ann Surg.

[7] Hyltander A, Liljegren E, Rhodin PH, Lönroth H (2002). The transfer of basic skills learned in a laparoscopic simulator to the operating room. Surg Endosc.

[8] Korndorffer JR, Dunne JB, Sierra R, Stefanidis D, Touchard CL, Scott DJ (2005). Simulator training for laparoscopic suturing using performance goals translates to the operating room. J Am Coll Surg.

[9] Sroka G, Feldman LS, Vassiliou MC, Kaneva PA, Fayez R, Fried GM (2010). Fundamentals of laparoscopic surgery simulator training to proficiency improves laparoscopic performance in the operating room-a randomized controlled trial. Am J Surg.

[10] Atesok K, Mabrey JD, Jazrawi LM, Egol KA (2012). Surgical simulation in orthopaedic skills training. J Am Acad Orthop Surg.

[11] Eden J, Berwick DM, Wilensky GR, Committee on the Governance and Financing of Graduate Medical Education, Board on Health Care Services, Institute of Medicine (2014). Graduate Medical Education That Meets the Nation’s Health Needs.

[12] Thomas GW, Johns BD, Marsh JL, Anderson DD (2014). A review of the role of simulation in developing and assessing orthopaedic surgical skills. Iowa Orthop J.

[13] Chapek M, Otlans PT, Buuck T (2024). Resident performance on the Fundamentals of Arthroscopic Surgery Training workstation does not predictably improve with postgraduate year. Arthrosc Sports Med Rehabil.

[14] Palter VN, Grantcharov T, Harvey A, Macrae HM (2011). Ex vivo technical skills training transfers to the operating room and enhances cognitive learning: a randomized controlled trial. Ann Surg.

[15] Tronchot A, Casy T, Vallee N (2023). Virtual reality simulation training improve diagnostic knee arthroscopy and meniscectomy skills: a prospective transfer validity study. J Exp Orthop.

[16] Gallagher AG, O’Sullivan GC (2011). Fundamentals of Surgical Simulation: Principles and Practice.

[17] Pedowitz RA, Nicandri GT, Angelo RL, Ryu RKN, Gallagher AG (2015). Objective assessment of knot-tying proficiency with the Fundamentals of Arthroscopic Surgery Training program workstation and knot tester. Arthroscopy.

[18] Farooq H, Gaetano A, Salazar D, Garbis N (2025). Guided education and the Fundamentals of Arthroscopy Surgery Training (FAST) workstation improve surgical resident knot-tying skills. Arthrosc Sports Med Rehabil.

[19] Pedowitz R, Nicandri G, Tuchschmid S (2016). Asymmetry in dominant / non-dominant hand performance differentiates novices from experts on an arthroscopy virtual reality serious game. Stud Health Technol Inform.

[20] Stefanidis D, Acker CE, Swiderski D, Heniford BT, Greene FL (2008). Challenges during the implementation of a laparoscopic skills curriculum in a busy general surgery residency program. J Surg Educ.

