# Supplementary material for: Benchmark Development for Fundamental Arthroscopic Skills Using a Simulation-Based Training Program: Observational Study
Source: JMIR Perioper Med. 2026 Apr 8;9:e82723. doi: 10.2196/82723 (PMC13060741; doi:10.2196/82723)

# Multimedia Appendix

This Multimedia Appendix provides detailed procedural descriptions of the five simulation-based arthroscopic skills modules evaluated in this study. These details are included to support reproducibility and implementation, while the main manuscript focuses on study design, benchmark derivation, and results.

## Probing:

During the probing task, the subject is instructed to identify and probe 10 of the numbers on the module. These are provided by the proctor in a randomly determined sequence (Figure 1) as determined by a random number generator [10]. 10 numbers were generated from a lower limit of 1 and upper limit of 21. No duplications were allowed, and the results were not sorted and were displayed as integers. Probing an incorrect number of results in failure of the module.

**Figure 6. Probing Testing Module**


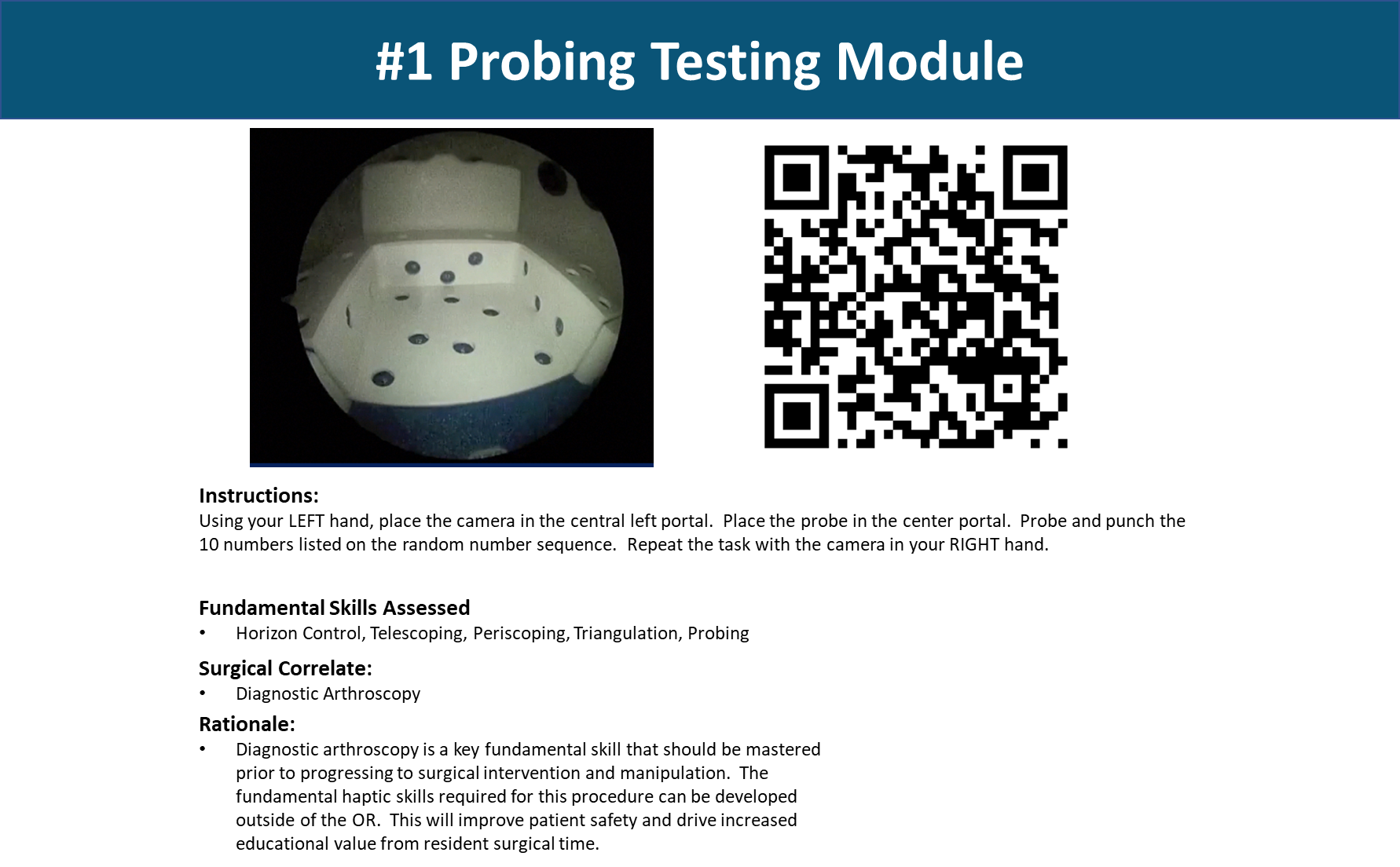


## Ring Transfer:

During the ring transfer task, the subject is instructed to transfer 8 rings from pegs on one side of the module to the pegs on the other side and then back again (Figure 2). The rings are initially placed on the opposite side of the working portal. For example – if the working portal (the one used to insert the ring grasping instrument) is on the right, the rings are placed on the left side of the module. If the subject drops a ring they are asked to continue the task. The number of dropped rings indicates the number of errors recorded.

**Figure 7. Ring Transfer Module**


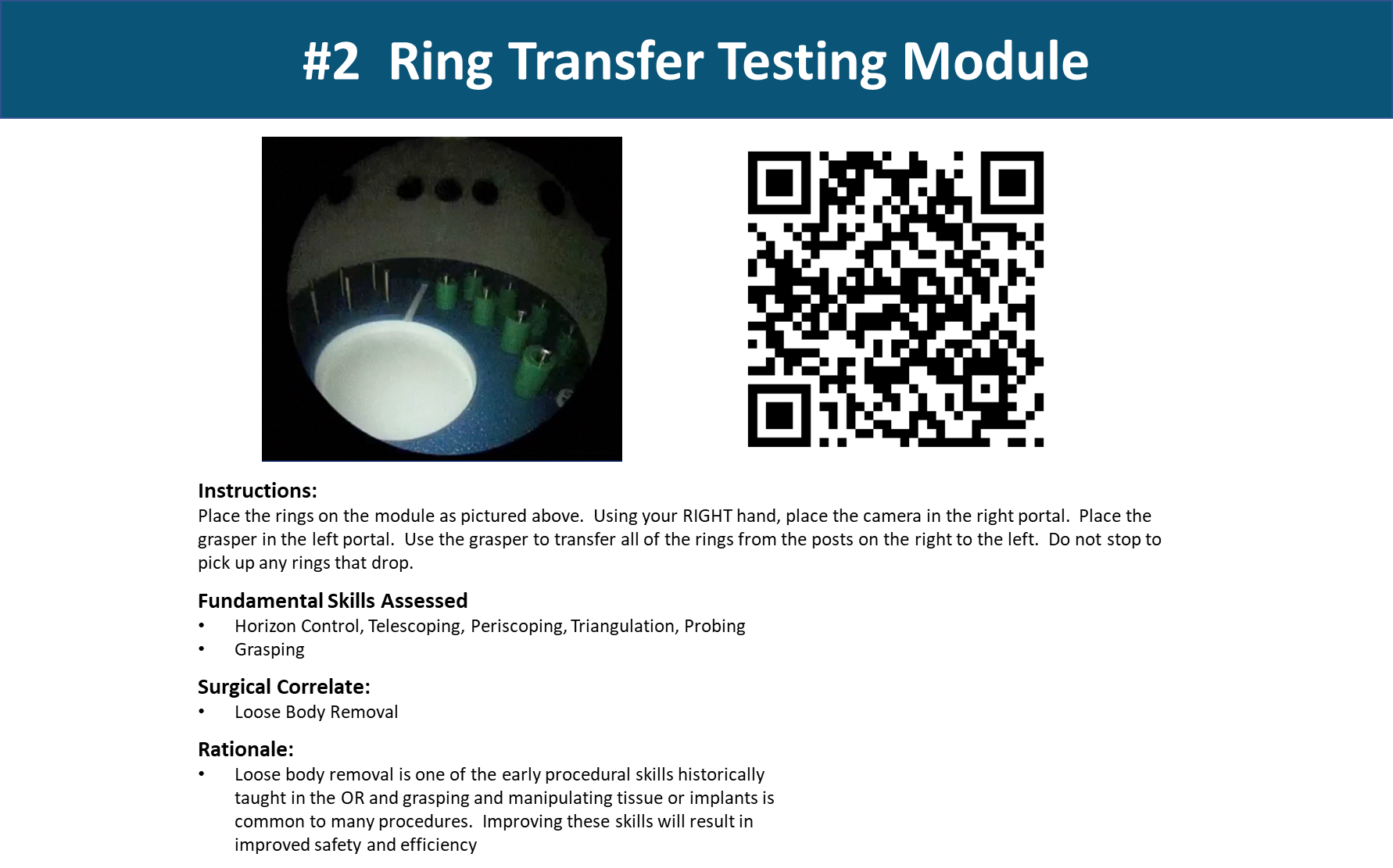


## Maze:

During the maze task, the subject is asked to use an arthroscopic probe to push a 3mm ball bearing though the maze (Figure 3). If the ball skips the track but stays on the platform, the subject may continue. If the ball jumps off the platform completely, this results in failure of the module.

**Figure 8. Maze Testing Module**


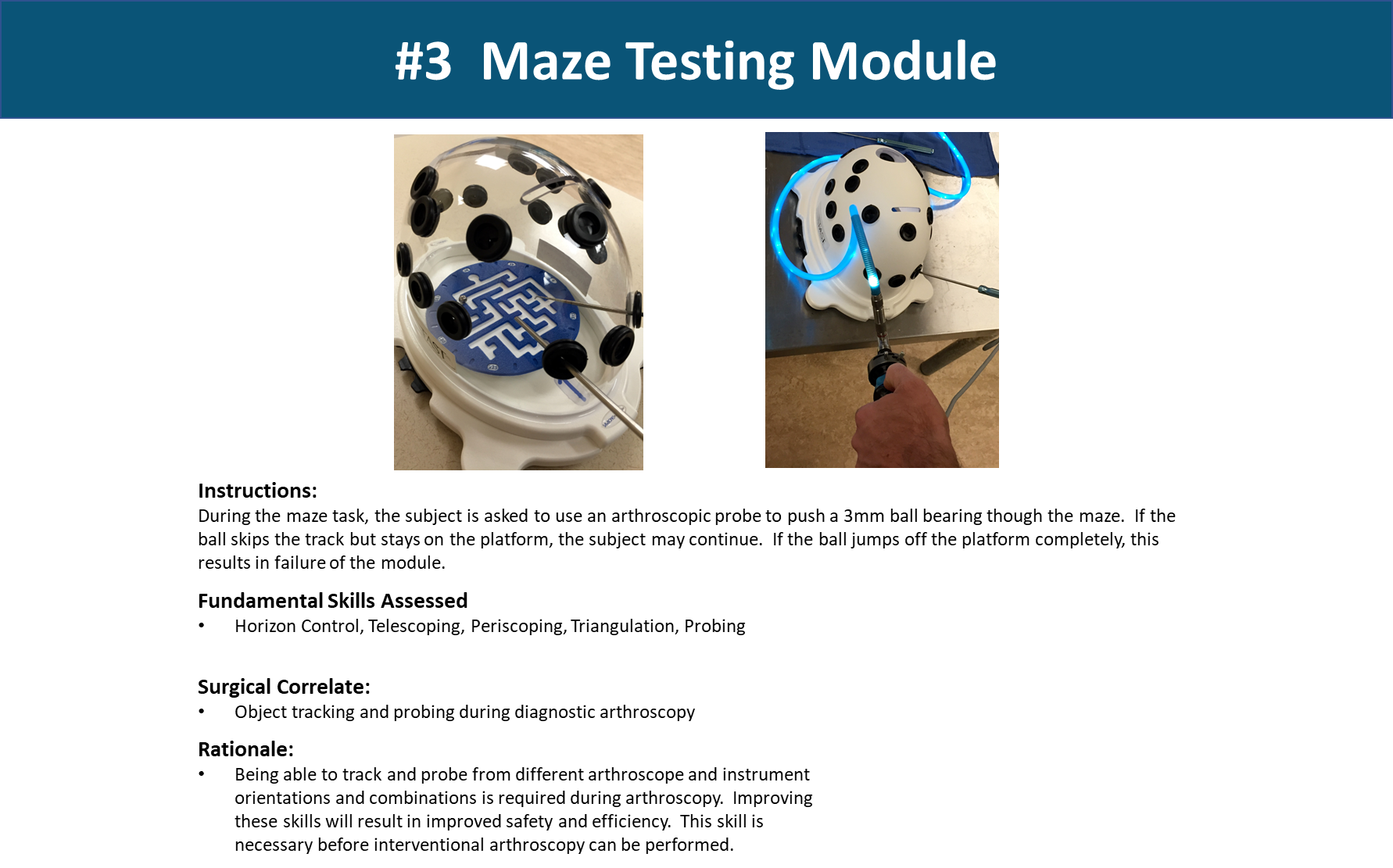


## Meniscectomy:

During the meniscectomy task, the subject is instructed to resect a paper “meniscus” template by removing all of the inner black line while leaving a complete outer black line margin (Figure 4). Failure to remove any part of the inner black line is called under-resection. Penetrating any part of the outer black line is called over-resection. Both under and over resection are counted as errors. Each instance is recorded as a separate error.

**Figure 9. Meniscectomy Module**


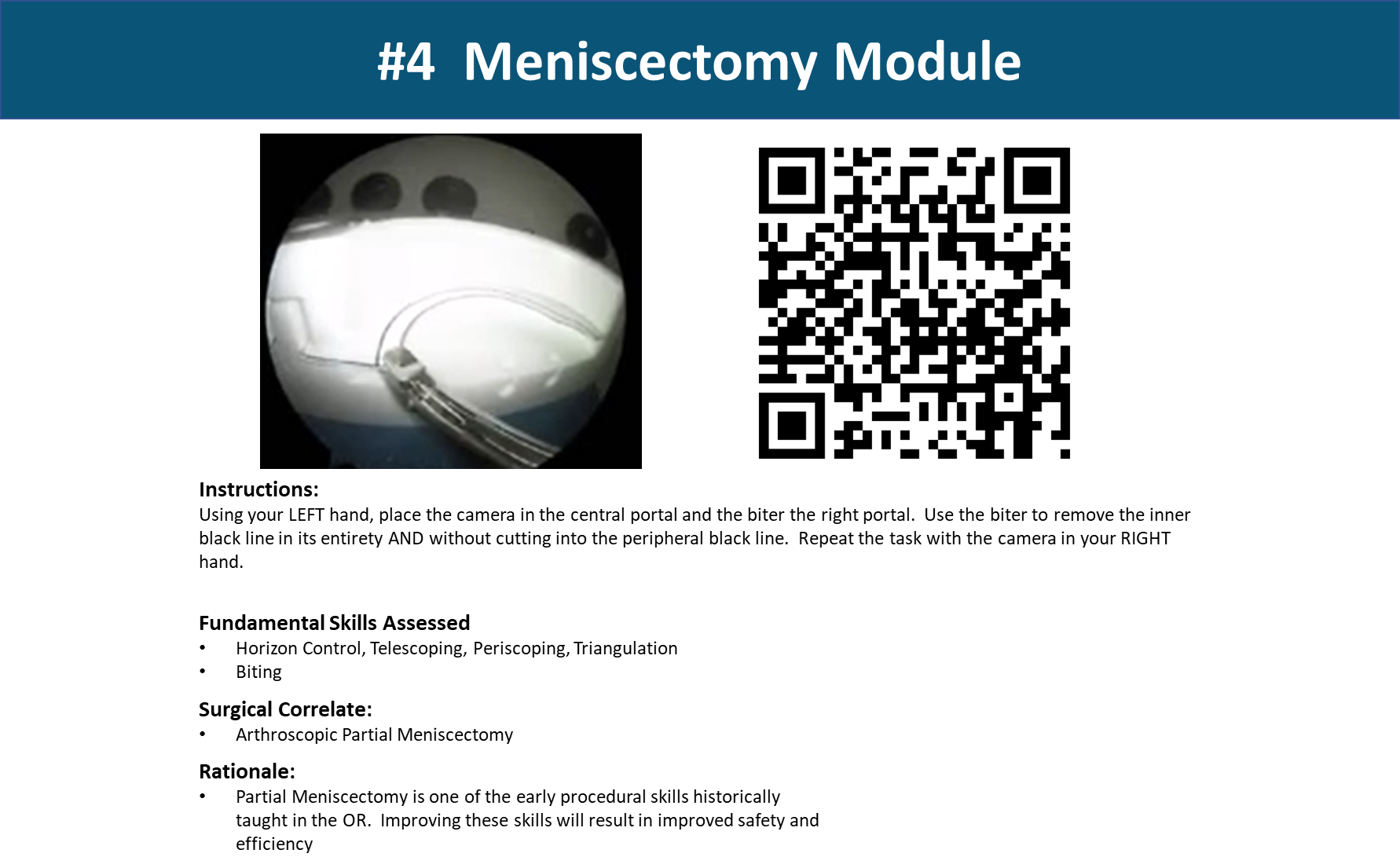


## Suture Passing:

During the suture passing task, the subject is instructed to use an antegrade suture passing device to pass three suture limbs from three previously loaded eyelets through the simulated rotator cuff as close to the pre-marked “targets” as possible (Figure 5). After passage, distance of the suture from the target is measured in millimeters and the total distance of all three sutures from their respective targets are recorded. A distance of more than 1mm of the pre-marked targets is considered an error. Unloading a suture from the eyelet results in failure of the module.

**Figure 10. Suture Passing Module**


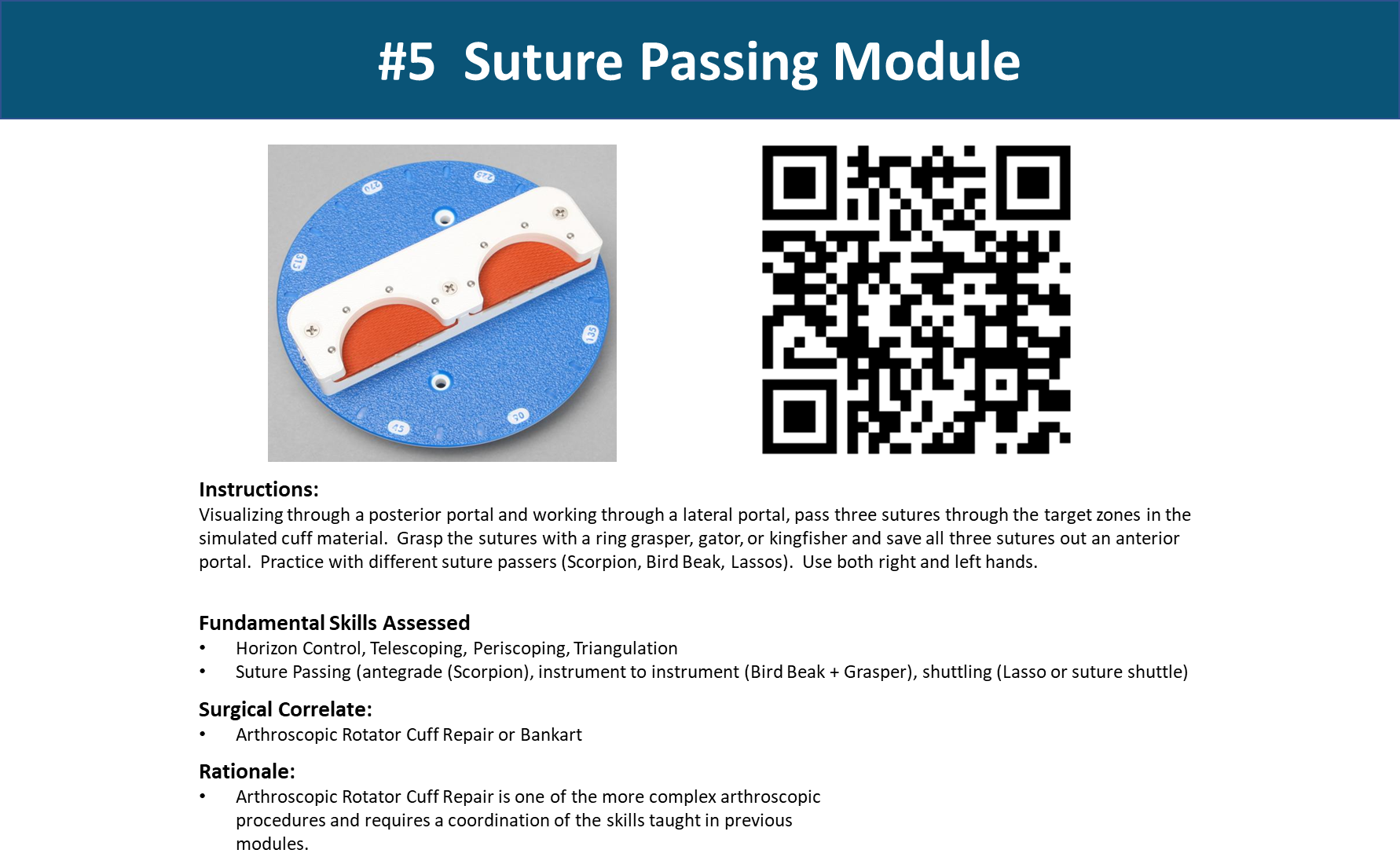

Supplement: Multimedia Appendix 1 [file periop-v9-e82723-s001.docx]
